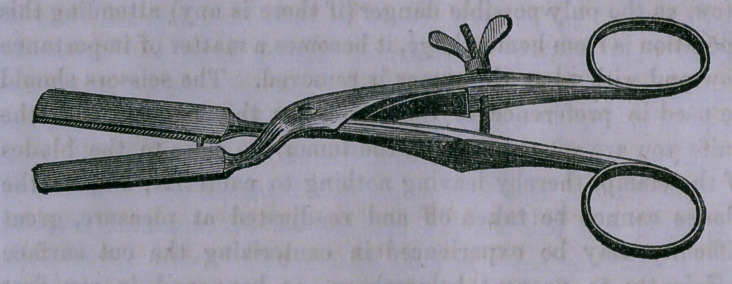# Observations on the “Clamp” in the Treatment of Internal Hemorrhoids and Prolapsus of the Rectum, with Two Cases

**Published:** 1866-12

**Authors:** A. J. Baxter

**Affiliations:** Chicago, Ill.


### *Observations on the "Clamp," in the treatment of Internal Hemorrhoids and Prolapsus of the Rectum—With two Cases.* By A. J. Baxter, M. D., Chicago, Ill.

The frequency of hemorrhoidal affections, the great distress which they occasion, and their liability to undermine the constitution, when of long duration, make them an important subject for surgical attention.

Heretofore the methods of treating internal hemorrhoids have been principally three, viz: Excision, nitric acid and the ligature—but from the excessive hemorrhage attending the first of these methods, the great difficulty in arresting it, and the fact that it has been attended with death in the hands of such men as Sir Astley Cooper, Dupuytren, and others of less note, have rendered it, I may say, obsolete. As the pathology of hemorrhoids became better understood, so the application of nitric acid became restricted, until now its usefulness is recognized in but one form of piles—the vascular excrescence, it being, as the name implies, a florid, granular, excessively vascular and slightly raised condition of the mucous membrane; ranging in size from a five cent piece to a quarter—most frequently of an intermediate size. This is the reason why nitric acid has met with so little favor at the hands of the profession. It has been used indiscriminately in all the varieties of piles, and being applicable to but one, resulted in a failure in the majority of cases. The advantages claimed in the ligature are simplicity of execution, safety from hemorrhage, certainty of result, and in the main, freedom from subsequent danger. If these advantages were not attended by any disagreeable consequences, they would be all that could reasonably be desired—but there are disadvantages and inconveniences attending its use of no miner importance, such as the length of time required for the separation of the ligature—ranging from five days to two weeks, according to the amount of bowel included; the pain and constitutional irritation often accompanied by difficulty in voiding urine, sometimes amounting to retention; and that its use is sometimes attended with death from pyæmia there is no doubt.

With this much in regard to the various methods of dealing with internal hemorrhoids, their advantages and disadvantages,

I will introduce the "Clamp" as improved by Henry Smith, Assistant Surgeon to King's College Hospital, London; and as I am not claiming anything original in this matter, but simply desire to call the attention of the profession to the great improvement afforded by this instrument in the treatment of internal hemorrhoids, I will give Mr. Smith's description of it, mode of application, etc.: "The instrument is much the same as regards shape as that originally recommended by Mr. Curling; but the edges of the blades, instead of being serrated, fit into one another by means of a groove and raised surface; and instead of being fixed by a catch, the handles are perforated by a light screw, so that the pressure can both be put on and taken off the tumor to be removed in such a gradual manner that any bleeding point may be readily appreciated and treated. The operation consists in seizing the tumor with the blades of the clamp or forceps, and removing it with a knife or sharp scissors; the raw surface is then wiped dry and thoroughly cauterized, either by nitric acid or the hot iron, according to circumstances; the parts are well oiled, and the affair is finished. The disease is removed at once, and the patient is not subjected to the irritation and danger of a ligature strangulating several portions of mucous membrane for a week or more. A very extensive experience of this method of treatment has convinced me so entirely in its superiority over every other plan, that I have thought it right to detail a few of the most severe cases which I have met with during the last year.

"I am the more anxious about this, as there is a feeling of prejudice against the treatment amongst those who have not witnessed it. On the other hand, I believe all those who have seen the operation as I adopt it in King's College Hospital, and in my private practice, have been much astonished and pleased with the results. My own impression is, that the ligature may be almost entirely done away with for hemorrhoids and prolapsus; although my own practice is somewhat contrary to my convictions, as I have latterly employed the ligature in a considerable proportion of cases which have occurred to me. The reason of this is, that I have been testing with great care the comparative value of the two modes of treatment, and have been selecting cases for each; and as before stated, the conclusion is forced upon me that the advantages of the plan by the clamp are too marked to be lost sight of."

My experience with the clamp, though nothing in comparison with Mr. Smith's, fully sustains all he claims for it, as I think the following cases will bear witness:

CASE I. Mrs. R——, aged twenty-one, a highly cultivated and sensitive lady—has been married but six weeks, says she has suffered more or less from piles during the last three years, but not so intensely until within the last six months, when she first noticed that something "came down." Ever since which she has been losing a good deal of blood, and the pain attending defecation has been greatly increased. Thinks they were brought on by constipation, from which she suffers habitually. May 20th I removed one large vascular tumor by means of the clamp and hot iron. One external pile was snipped off with the scissors. On the third day her bowels were moved with a castor oil emulsion, and on the fifth day she and her husband called at my office. Complained only of some soreness when the external pile was removed.

CASE II. Mrs. G——, aged thirty-five, a stout, hearty Irish washerwoman—mother of three children, sent for me, Tuesday morning, September 4th. I found her in bed, with a high fever, and complaining of great pain and burning in her "behind." On examination, found two large tumors, one of which was in a high state of inflammation, partially protruding, and tightly grasped by the sphincter. The tumors were removed in the afternoon, with the aid of the clamp and hot iron. To allay the constitutional trouble and quiet pain, directed that a tablespoonful of the following solution should be given every hour until three doses were taken, then to be given once in two hours: ℞ Liq. ammoniæ, acet. ℥vi., morph. acetat. gr. iss. Cautioned her particularly to keep quiet in bed.

5th. Found her in bed, fever nearly gone. Expressed herself as feeling perfectly well. Directed her to remain in bed until the following day, when, if everything continued favorable, she might get up. On calling the next day, to my astonishment, I

found her at the washtub. Gave her some castor oil and departed. This case fully illustrates, I think, the advantage of the clamp over the ligature. The most zealous advocate of the latter would honestly have thought of applying it here, but would have put in force measures for subduing the local and constitutional trouble, such as leeches, hot water dressings or poultices, thereby consuming more time and the enduring of more pain in palliative treatment than was required for the radical cure.

Mr. Smith, in describing the operation, says the tumor is seized with the clamp and removed with a knife or scissors. Now, as the only possible danger (if there is any) attending this operation is from hemorrhage, it becomes a matter of importance how and with what the tumor is removed. The scissors should be used in preference to the knife, for this reason: with the knife you are apt to shave off the tumor too close to the blades of the clamp, thereby leaving nothing to cauterize, and as the blades cannot be taken off and readjusted at pleasure, great difficulty may be experienced in cauterizing the cut surface sufficiently to prevent hemorrhage, as happened in my first operation, causing no little trouble and anxiety. And again, it is a matter of no small importance whether nitric acid or the hot iron is used as the cauterizing agent. Mr. Smith says, "When I first began to use this treatmeat, I employed nitric acid as the cauterizing agent in all cases; but in two instances it failed to stop the bleeding, and I was compelled to secure the part with a ligature. Consequently, I now only use the nitric acid in the slight cases; but if the diseased mass be extensive, or the parts extraordinarily vascular, I employ the actual cautery, which suffices to seal up the vessels. I am inclined to think that, of the two, the cautery is followed by more suffering than when nitric acid is used. Admitting that a little more suffering attends the use of the cautery, no judicious surgeon would, I think, jeopardize the safety of his patient to avoid a little additional suffering; and as an anodyne sufficient to allay pain should follow either application, the objection amounts to nothing, whereas the certainty of the cautery in preventing hemorrhage."

After giving the details of seventy cases in which the clamp had been used, Mr. Smith draws the following conclusions: 1st, That it is safer than the ligature, for in no one case has death occurred, nor in any one instance has anything taken place which caused the least anxiety; 2d, That it is less painful, and followed by less constitutional irritation; 3d, That it is much quicker—the patient not being confined to bed more than one-third the time necessary for the separation of the ligature; and, 4th, The result is as lasting as that by the ligature, because in each instance the disease is bodily removed—in one case speedily; in the other slowly.

The clamp can be obtained of Messrs. Bliss & Sharp, 144 Lake street, in this city.

## Figures and Tables

**Figure f1:**